# A Physics-Informed Neural Network for *In Vivo* Dosimetry Using Quantitative Radiacoustic Imaging

**DOI:** 10.21203/rs.3.rs-8503498/v1

**Published:** 2026-01-20

**Authors:** Leshan Sun, Kristina Bjegovic, Lucia Rodriguez-Gonzalez, Yifei Xu, Yuchen Yan, Gilberto Gonzalez, Lucy Whitmore, Luke Connell, Yankun Lang, Prabodh Pandey, Lei Ren, Emil Sch¸ler, Yong Chen, Shawn Xiang

**Affiliations:** 1 The Department of Biomedical Engineering, University of California, Irvine, Irvine, United States; 2 The Department of Radiological Sciences, University of California, Irvine, Irvine, United States; 3 Beckman Laser Institute & Medical Clinic, University of California, Irvine, United States; 4 Department of Radiation Oncology, University of Oklahoma Health Sciences Center, Oklahoma city, OK, United States; 5 Department of Radiation Physics Division of Radiation Oncology, The University of Texas MD Anderson Cancer Center, Houston, TX, United States; 6 UTHealth Houston Graduate School of Biomedical Sciences, The University of Texas MD Anderson Cancer Center, Houston, TX, United States; 7 Department of Radiation Oncology, University of Maryland School of Medicine, Baltimore, MD, United States

**Keywords:** Radiotherapy, *In Vivo* Dosimetry, Quantitative Radiacoustic Imaging, Physics-informed Neural Network

## Abstract

Accurate dosimetry is critical for safe and effective radiotherapy, yet no clinical method currently measures dose directly within the patient *in vivo*. Radiacoustic imaging (RAI), which detects acoustic waves generated by thermoelastic expansion during radiation delivery, offers a promising solution but has been limited to qualitative output. We present a quantitative RAI (qRAI) framework powered by a physics-informed neural network (PINN) that reconstructs quantitative dose maps *in vivo*. The PINN incorporates the physics of acoustic wave generation and propagation, along with a digital twin of the radiation delivery and radiacoustic detection systems, enabling accurate reconstruction from limited-view data. Reconstructed pressure maps are calibrated against experimental and simulated dose references. We validate the method across diverse clinical scenarios, including water tank dosimetry, human torso phantoms, and FLASH electron therapy. Compared to purely data-driven models, our PINN approach offers superior robustness and generalizability, especially in clinical settings lacking experimental ground truth. These results establish PINN-based qRAI as a powerful tool for real-time, adaptive, and quantitative *in vivo* dosimetry.

## Introduction

Cancer remains one of the leading causes of death globally, with radiotherapy playing a central role in treatment—used in over half of all cancer cases^1,2^. As treatment technologies evolve, advanced modalities such as proton therapy^3^ and FLASH^4^ radiotherapy have gained prominence due to their ability to improve tumor targeting while minimizing damage to healthy tissues. FLASH radiotherapy^5,6^ delivers radiation at ultra-high dose rates (≥40 Gy/s), with instantaneous rates reaching up to millions of Gy/s, and proton therapy^7,8^ uses charged particles to deposit energy precisely at the Bragg peak, reducing exposure beyond the tumor. The clinical adoption of proton therapy is accelerating rapidly, with treated patient numbers tripling between 2012 and 2021, and over 110 centers now in operation globally^9^. A recent milestone in accessibility was marked by Stanford’s installation of the first compact Proton Therapy System^™^ in a standard LINAC vault^10^, indicating a scalable path to broader clinical use. As these advanced radiotherapies gain traction, the need for accurate *in vivo* dosimetry—the ability to measure the delivered dose in real time inside the patient—becomes critical to ensuring both treatment efficacy and patient safety^11–13^.

Yet, no existing clinical method enables direct, real-time *in vivo* measurement of radiation dose^14,15^. Current techniques rely on pre-treatment planning CT and external detectors like ion chambers^16,17^ or radiochromic films^18–20^, which cannot capture dose variations during treatment. Alternatives such as PET^21–23^ and prompt gamma imaging^24–27^ offer limited utility due to low detection efficiency, delayed signal acquisition, and complex system integration^28^. Cerenkov imaging provides promising surface dose verification but lacks the ability to resolve deep tissue dose in three dimensions^29–31^. These limitations highlight the urgent need for a real-time, 3D volumetric dosimetry method capable of verifying delivered dose directly on patients during radiotherapy^13^.

Radiacoustic imaging (RAI) has recently emerged as a promising solution for this unmet need^32–39^. RAI detects acoustic waves generated by rapid thermoelastic expansion of irradiated tissues, enabling non-invasive dose visualization with ultrasound detectors^40^. A major milestone was achieved with real-time 3D visualization of radiation dose in liver cancer patients using clinical linear accelerators^41^. RAI has been demonstrated with X-rays^42^, electrons^43^, and proton beams^37^. However, most existing RAI image reconstructions rely on simple back-projection algorithms^44^, which assume idealized imaging conditions and fail under practical constraints such as limited-view acquisition, noise, and finite transducer bandwidth^45–48^. These algorithms produce artifacts, including negative pixel values that invalidate physical dose interpretation^49^. While model-based iterative reconstruction methods can yield higher-fidelity images by incorporating accurate physical models and regularization, they are computationally intensive and not feasible for real-time applications in clinical settings^50–53^.

To address these challenges, deep learning-based reconstruction has shown promise in recovering accurate images from limited-view data^54–61^. Neural networks can learn inverse mappings that correct artifacts and recover dose distributions efficiently^62–64^. However, purely data-driven approaches require large and diverse training datasets^65–67^—which are currently unavailable for RAI due to its novelty. Furthermore, generating ground truth for network training is nontrivial, as the initial pressure distribution inside biological tissues is experimentally inaccessible^14,15^.

**Figure 1.**
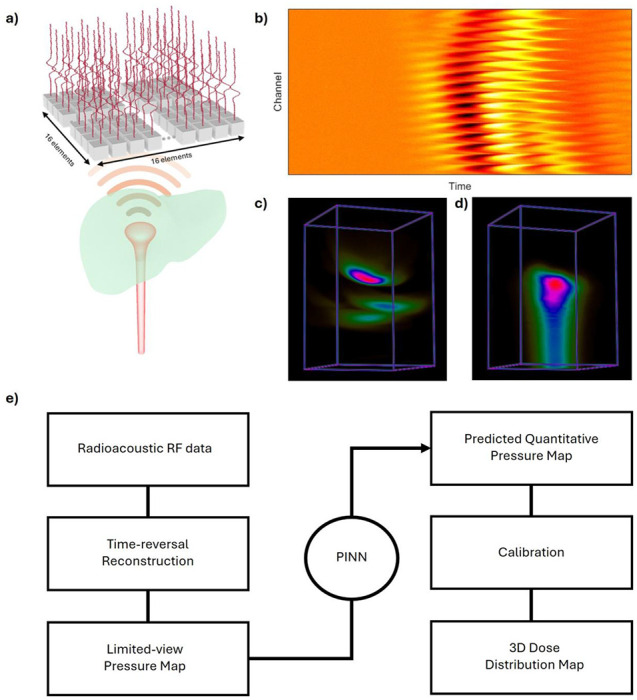
Schematic figure for *in vivo* dosimetry with qRAI. a) The clinical implementation demonstration of qRAI. The radiation beam will be delivered to the tumor (liver tumor for demo). Then the generated qRAI signals will be captured by implementing an US transducer array. b) The acquired qRAI radiofrequency sinogram from the US transducer array. c) The corresponding limited-view dose reconstruction. d) The PINN enhanced quantitative dose map. e) The workflow of the radiacoustic imaging. The RAI radiofrequency data will be acquired during the radiotherapy and then reconstructed using time-reversal reconstruction methods. The reconstructed limited-view pressure map will be input into the PINN for enhancement. The predicted quantitative pressure map was then converted to 3D dose distribution map using calibrated dose coeffcient.

To overcome these limitations, we introduce a quantitative RAI (qRAI) framework powered by a physics- informed neural network (PINN) in Fig.1. In this framework, the radiation beam is delivered to the targeted area, and the resulting qRAI signals are captured by an ultrasound (US) transducer array, forming a radiofrequency sinogram (Fig. 1b). This sinogram is then reconstructed using a time-reversal algorithm68 resulting in a limited-view dose map (Fig. 1c). To improve this reconstruction, we apply a PINN-enhanced model, yielding an enhanced dose map (Fig. 1d). The complete workflow of the radiacoustic imaging process is illustrated in Fig. 1e and will be further detailed in subsequent sections. Our PINN incorporates the underlying physics of acoustic wave generation and propagation, along with digital twins of the radiation delivery and ultrasound detection systems. This hybrid approach enables robust reconstruction of quantitative dose map from limited-view measurements, without the need for extensive ground-truth datasets. Unlike traditional deep learning, PINNs integrate governing physical equations directly into the training process, resulting in models that are more data-efficient, interpretable, and generalizable^69–73^. Specifically, our framework includes a general radiacoustic model for wave generation and propagation, a transducer twin capturing system-specific characteristics (geometry, aperture, impulse response), and a radiation beam twin created using a treatment planning system or TOPAS to simulate dose deposition and temporal pulse profiles. These components guide the PINN training through both forward and inverse models, enabling accurate image reconstruction under clinically relevant conditions. We validate the proposed system using proton and electron beam experiments in water phantoms and a human torso phantom, demonstrating the feasibility of real-time, *in vivo* radiation dosimetry with potential to significantly advance the precision and adaptability of next-generation radiotherapy.

## Results

### Framework of PINN

**Figure 2.**
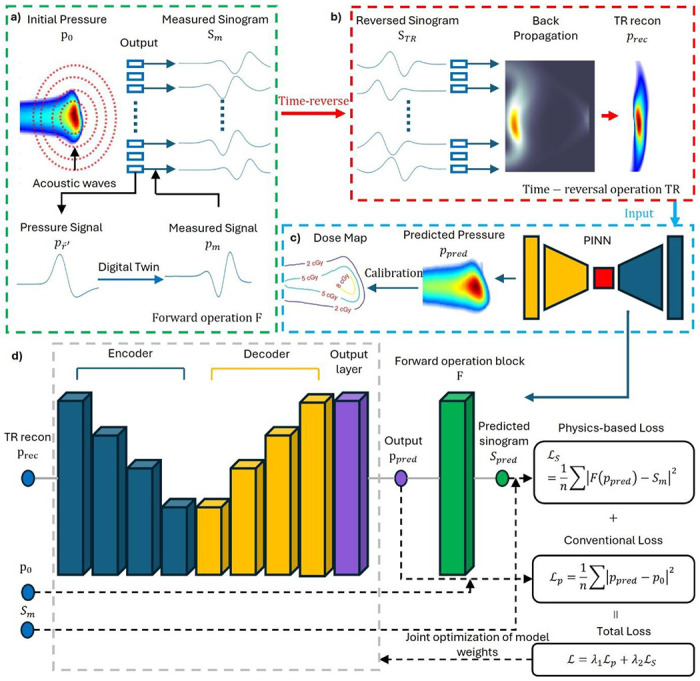
Complete workflow of PINN implementation. a) The forward operation of PAT. Radiation beams generate initial pressure *p*_0_ and the acoustic waves will propagate spherically through the medium. The arrived pressure signals $p{\vec{r}}^{\prime }$ are captured by the transducer at different time as measured signals *p*_*m*_, where the process are modeled as a digital twin system, to form the measured sinogram S_*m*_. b) The inverse operation of PAT. S_*m*_ is reversed in time to get the time-reversed sinogram *S*_*TR*_. The signal was then backpropagate to the medium and converge to time-reversal reconstruction *p*_*rec*_. c) Converting limited-view pressure map to dose map. The time-reversal reconstruction will be input to the PINN for enhancement and then go through a calibration procedure to convert to dose. d)The workflow of PINN. Three datasets, *p*_*rec*_, *p*_0_, and S_*m*_ are input into the network. The output *p*_*pred*_ is used to generate predicted sinogram *S*_*pred*_ to calculate the physics based-loss *ℒ*_*s*_. Then the total loss was calculated from *ℒ*_*s*_ and conventional loss *ℒ*_*p*_ to function as a joint optimization for the model training.

We developed a physics-informed neural network (PINN) to enhance limited-view qRAI, as illustrated in Fig. 2. Unlike conventional deep learning approaches that require large, labeled datasets, our PINN integrates physical models directly into the learning process, enabling robust reconstruction from limited experimental data. There are four major components: a) Forward Operation of radiacoustic imaging (RAI). As shown in Fig. 2a, radiation dose deposition induces an initial pressure rise, *p*_0_, which generates spherically-propagating acoustic waves. These pressure signals, $p{\vec{r}}^{\prime }$, are detected by an ultrasound transducer array over time, forming the measured sinogram S_*m*_. This forward process is modeled using a digital twin system that incorporates: the radiation pulse profile, acoustic propagation through heterogeneous media, transducer impulse response and geometry via finite-element modeling. This comprehensive forward operator *F* provides the physical basis for accurate modeling of the qRAI data acquisition process. b) Inverse Operation: Time-Reversal Reconstruction. As shown in Fig. 2b, the inverse process is approximated via time-reversal (TR) reconstruction^68^. The measured sinogram S_*m*_ is reversed in time to obtain S_*TR*_, which is then backpropagated through the medium to yield a reconstructed pressure 110 map *p_rec_*. However, this TR-based inverse operator does not yield a unique solution under limited-view conditions^55^, which is a major limitation in clinical settings. Our prior work^63^ addressed this issue using a U-Net architecture for post-processing and a direct reconstruction approach^62^, both validated in simulation only due to the lack of experimental datasets. c) Dose Mapping via PINN Enhancement. To overcome the limited-view artifacts and derive quantitative dose maps, we introduce a PINN (see Fig. 2c). The TR reconstruction *p_rec_* is enhanced by the PINN to yield a predicted pressure map *p_pred_*, which is then calibrated into dose map using experimentally determined coefficients (detailed in Methods). d) PINN Architecture and Physics-Based Loss. The PINN architecture is based on a 3D U-Net (Fig. 2d), designed to map *p_rec_* to the initial pressure *p*_0_. To embed physical consistency, we incorporate a non-trainable forward operator module *F* into each training iteration: The enhanced prediction *p_rec_* is passed through *F* to generate the predicted sinogram *S_pred_*. A physics-based loss *ℒ_S_* is computed between *S_pred_* and the actual measured sinogram S_*m*_. A conventional loss *ℒ_p_*, comparing *p_rec_* to *p_rec_*, is also computed. The total loss is then defined as a weighted combination: *L*=*λ_1_ℒ_p_*+*λ_1_ℒ_S_*, where *λ_1_* and *λ_2_* are tunable weighting factors. This joint optimization enforces both data fidelity and physical plausibility, enabling the model to generalize well in the absence of large-scale training data—a key advantage for this emerging imaging modality.

### Improved Signal Fidelity with Digital Twin

**Figure 3.**
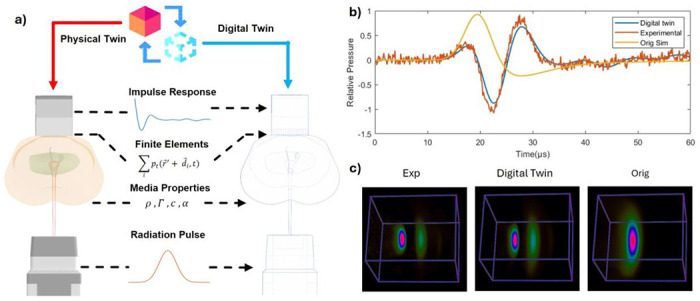
Demonstration and results of the Digital Twin system. **a)** Illustration of the digital twin system. Radiation beams come with a temporal pulse profile that will affect the initial pressure. The medium is defined based on the material to take density, Gruneisen Parameters, speed of sound, and attenuation into consideration. The transducer element was modeled to take the finite elements effects into consideration. And finally, the impulse response from the PZT element was measured and involved in the system **b)** The demonstration of the simulated signal. The Yellow line is the original k-Wave simulation with only media taken into consideration. The red line shows the experimental signal in water tank, and the blue line shows the digital twin system simulated signal. **c)** Corresponding time-reversal reconstructions from the signals. The experimental one and the digital twin one is comparable, while the reconstruction from the original signal shows only one peak and enlarged area.

To enable accurate simulation of system-specific effects in qRAI, we developed a digital twin^74,75^ model of our experimental setup (Fig. 3a). This virtual replica captures the influence of both the radiation delivery system and the ultrasound detection hardware. Here, we present validation results demonstrating the improved fidelity of the digital twin simulation compared to conventional acoustic-only models. Fig. 3b compares three representative radiacoustic signals: the yellow line shows a standard k-Wave simulation^76,77^ that includes only medium properties and basic acoustic propagation, producing a single peak corresponding to the initial pressure response; the red line represents the experimentally measured signal from a water tank irradiated by a proton beam, which exhibits a distinct double-peak structure; and the blue line shows the digital twin simulation that incorporates radiation pulse profile, medium characteristics, transducer geometry, and measured impulse response—closely matching the experimental signal aside from minor electromagnetic interference. Fig. 3c further demonstrates that time-reversal reconstruction based on the experimental and digital twin signals both yield two localized peaks of comparable size and location, accurately reflecting the expected dose distribution. In contrast, reconstruction from the standard simulation fails to recover this structure, showing only a single, broadened peak. These results confirm that integrating system-specific effects through the digital twin significantly enhances agreement with experimental data in both temporal and spatial domains. Moreover, the high fidelity of the digital twin simulation supports its use as a reliable surrogate for generating training data in physics-informed or hybrid deep learning frameworks, addressing the current limitation of experimental data scarcity in emerging qRAI applications.

### Water Tank Evaluation in Proton Therapy

**Figure 4.**
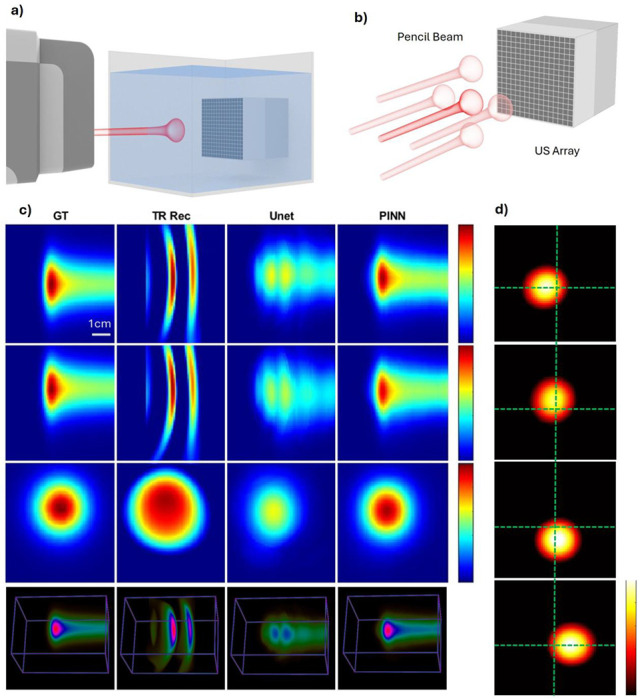
Proton water tank dose reconstruction. a) Illustration of proton water tank experiment setup. The proton beam is delivered to the water tank. And the US transducer array is placed directly inside the water tank, facing the proton beam. The initial beam was planned at the center of the transducer array. b) Experiment design. To account for off-center effects, we shift the transducer for 1cm in for directions, identical to shifting the beam. c) Comparison of the ground truth (GT, first column), time-reversal reconstruction (TR Rec, second column), U-Net enhancement (third column), and PINN enhancement (fourth column) across four views: axial view (first row), sagittal view (second row), coronal view (third row), and the 3D volume (fourth row). d) The reconstruction results of the off-center beams. From top to bottom are: left, up, down, right beams. The green dashed lines illustrated the off-center effects. The center of the green dashed line is the center of the reconstructed center beam.

To evaluate the performance of the PINN, we first conducted a validation study using experimental data acquired from a clinical proton beam irradiation in a water tank (Fig. 4a). A clinical proton therapy system (Hyperscan S250i, Mevion, USA) was operated in service mode to enable precise control of pencil beam energy and pulse number. The beam operated at a pulse repetition rate of 750 Hz, delivering approximately 8 picocoulombs of protons per pulse. Log files from the proton system were recorded and used for quantitative benchmarking. A 16×16 matrix ultrasound array (Doppler Tech Inc., Guangzhou, China) was positioned on the side opposite to the proton gantry without interference to the proton beam. Radiacoustic signals generated by the proton beam were captured by this matrix array, amplified, and digitized using a custom data acquisition (DAQ) system (Photosound Tech Inc., Houston, USA).

The proton beam was initially targeted at the center of the array, followed by four additional measurements with 1 cm transducer shifts in the left, right, up, and down directions to simulate off-axis scenarios (Fig. 4b). This yielded a dataset of 7 different proton beam energies captured at 5 transducer positions, resulting in 35 sinograms. Two datasets with severe electromagnetic interference (EMI) were excluded, leaving 33 usable sinograms. These were divided into 20 for training, 5 for validation, and 8 for testing. Due to the limited size of the experimental dataset, we augmented the training data with synthetic sinograms generated from clinical treatment plans using our validated digital twin model. Specifically, 50 pencil beams from a prostate cancer plan and 25 from a liver cancer plan were simulated to create high-fidelity training examples. Initial model performance was evaluated using only the simulated data (details provided in Supplementary Information I and Supplementary Fig. 4). To further improve robustness and suppress experimental noise, the water tank data were combined with the simulation dataset to form a joint training set.

Fig. 4c shows representative comparisons of four reconstruction methods—ground truth (GT), time-reversal reconstruction (TR Rec), U-Net enhancement, and PINN enhancement—across axial, sagittal, and coronal views, as well as full 3D volumes. Fig. 4d illustrates the reconstructions from off-center beam positions (left, up, down, right). The green dashed lines denote the beam center of the original central position, highlighting the spatial shifts and the ability of PINN to correct off-center distortions. We additionally evaluated performance under varying proton pulse numbers, and Supplementary Fig. 7 shows that the quantitative reconstruction faithfully recovers distributions from the summed signals. Together, these results validate the accuracy and generalizability of the PINN framework across both central and off-axis beam positions using real-world proton beam data. Quantitative analysis in Table 1 confirms that the PINN outperforms the U-Net across multiple metrics: it achieves a substantially higher structural similarity index, improved peak signal-to-noise ratio, and higher Gamma Index passing rate. These results demonstrate that integrating the forward physics model into the network makes the PINN a powerful tool for accurate quantitative dose reconstruction even with the presence of noise measurement.

### Human Torso Evaluation during Proton Therapy

To evaluate the PINN under conditions that closely mimic clinical scenarios, we performed a validation experiment using an adult human-torso phantom (True Phantom Solutions, Ontario, Canada), offering a more anatomically realistic model than the water tank setup. A planning CT scan of the phantom was acquired, and single pencil beam dose distributions were generated using a commercial treatment planning system (RayStation, RaySearch Laboratories, Stockholm, Sweden).

**Figure 5.**
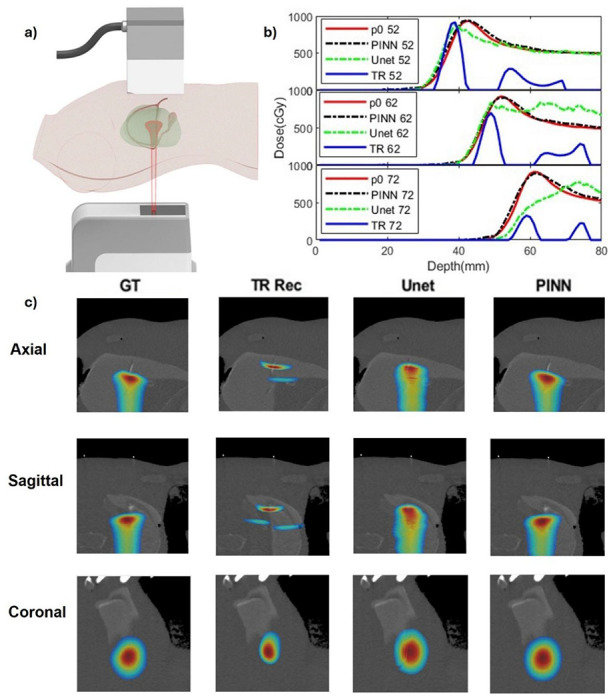
Proton human torso phantom dose reconstruction. a) Illustration of proton human phantom experiment setup. The proton beam is delivered to the liver area inside the human torso phantom. Three different proton energies (160.67, 165.95, 170.08MeV) were used to deliver the proton beam to different depths (52, 62, 72mm to the transducer array position). b) Reconstruction results for 3 different energies. Red solid lines show the initial dose; Blue solid line show the TR reconstruction; Black dashed line show the PINN results; Green dashed line show the Unet results. c) Comparison of the ground truth (GT, first column), time-reversal reconstruction (TR Rec, second column), U-Net enhancement (third column), and PINN enhancement (fourth column) across four views: axial view (first row), sagittal view (second row), and coronal view (third row). The results are overlayed with the phantom CT to better show the dose map distribution.

As shown in Fig. 5a, the proton beam was delivered from beneath the human torso, targeting the liver region, while a planar ultrasound transducer array was placed on the abdominal surface. The liver was chosen due to the presence of a favorable acoustic window in the abdomen and the absence of bone in the proton beam path, allowing for relatively homogeneous acoustic propagation. According to Supplementary Table 1, the acoustic properties of soft tissues and organs in this region are sufficiently similar to justify this assumption. Three distinct proton energies—160.67, 165.95, and 170.08 MeV—were used to deliver Bragg peaks at depths of 5.2 cm, 6.2 cm, and 7.2 cm from the transducer, respectively. Because this dataset included only three pencil beam energies, it was not sufficient for model retraining or fine-tuning. Instead, we directly applied the PINN model trained on simulated and water tank data to this new dataset. Fig. 5b presents line profile comparisons of the reconstructed dose distributions for all three energies. Time-reversal reconstruction (blue solid lines) consistently localized the Bragg peaks but failed to reproduce the correct amplitude and peak morphology. The U-Net predictions (green dashed lines) recovered amplitude more effectively, but introduced noticeable shape distortion, particularly for the 7.2 cm beam. In contrast, the PINN results (black dashed lines) closely matched the ground truth dose profiles (red solid lines), accurately capturing both shape and intensity across all energy levels.

Fig. 5c further visualizes dose reconstructions in axial, sagittal, and coronal views, overlaid on the planning CT. Compared to time-reversal and U-Net results, the PINN-enhanced reconstructions show superior spatial agreement with the planned dose distributions. These findings highlight the PINN’s ability to generalize to complex, real-world anatomies without retraining, thanks to its embedded physics-based forward model.

### Evaluation in FLASH Therapy

**Figure 6.**
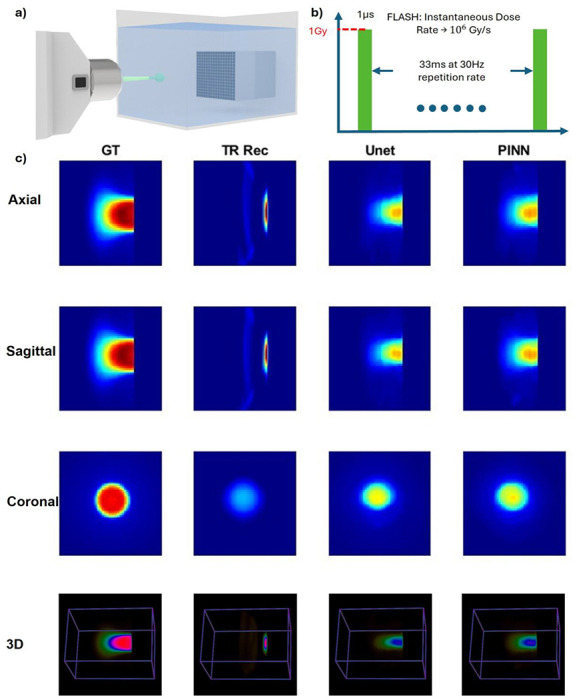
FLASH electron water tank dose reconstruction. a) Illustration of FLASH electron water tank experiment setup. The electron is shooting from MOBETRON machine to the water tank through a collimator. b) Illustration of FLASH dose rate. The FLASH electron beam can deliver 1Gy in 1μs, resulting in an instantaneous dose rate of 10^6^ Gy/s. c) Comparison of the ground truth (GT, first column), time-reversal reconstruction (TR Rec, second column), U-Net enhancement (third column), and PINN enhancement (fourth column) across four views: axial view (first row), sagittal view (second row), coronal view (third row), and 3D view (fourth row). The GT is extracted from the dose map generated from TOPAS. Both U-Net and PINN are tuned with new electron dataset generate with 6 different collimator settings

To further evaluate the versatility of PINN, we tested its performance using a different radiation modality—FLASH electron radiotherapy. As shown in Fig. 6a, the experiment was conducted in a water tank setup, through a collimator. This controlled configuration allowed us to capture dose distributions with high temporal resolution, making it ideal for testing qRAI’s real-time dosimetry capabilities under ultra-high dose rate conditions. A key characteristic of this experiment is the extreme dose rate of the FLASH electron beam, illustrated in Fig. 6b. The system delivers 1 Gy of dose in just 1 microsecond per pulse, corresponding to an instantaneous dose rate of 10^6 Gy/s. This mirrors the conditions of future clinical FLASH therapy^78,79^, where precise *in vivo* dosimetry remains a major technical challenge due to the rapid dose deposition.

To benchmark performance, we compared the reconstruction results of four methods (Fig. 6c): ground truth (GT), time-reversal (TR) reconstruction, U-Net enhancement, and PINN enhancement, across axial, sagittal, coronal, and 3D views. The GT dose maps were obtained using TOPAS^80^ Monte Carlo simulation, serving as the reference standard (Summary statistics of the training dataset are provided in Supplementary Figure 6 and Supplementary Table 2). Both U-Net and PINN models were fine-tuned via transfer learning using a new FLASH electron dataset that included six different collimator configurations to introduce variability and improve generalization (training details provided in Supplementary Information III).

The reconstruction results show that the TR method can roughly localize the dose but lacks structural accuracy. The U-Net model recovers some spatial features but performs inconsistently across different views and energy levels. The PINN model shows modest improvements in both spatial fidelity and intensity reconstruction; however, it still exhibits limitations in capturing the full dose structure. These challenges are largely attributed to the limited size and variability of the training dataset under FLASH conditions.

Despite these constraints, the experiment demonstrates the feasibility of extending PINN empowered qRAI and the framework to FLASH therapy applications. With further optimization and larger training datasets, this approach holds promise for accurate, real-time *in vivo* dosimetry in ultra-high dose rate radiotherapy.

## Discussion

We introduce a physics-informed neural network (PINN) framework for radiacoustic imaging (RAI), enabling quantitative, real-time, volumetric dosimetry during radiotherapy. Our results demonstrate that PINN offers several critical advantages over conventional reconstruction approaches—most notably, the ability to reconstruct accurate, quantitative dose maps from limited-view data without requiring labeled *in vivo* ground truth during treatment. This capability represents a major step towards *in vivo* dosimetry, where dose delivery can be verified in real time, even under clinical constraints.

A major challenge for deep learning in novel imaging modalities is the scarcity of large-scale annotated datasets. PINN directly addresses this by dramatically reducing training data requirements. Rather than relying on patient images, PINN is trained using synthesized sinograms generated from digital twins, which simulate a wide range of dose distributions and anatomical features. This not only eliminates the need for time-consuming and costly data collection but also mitigates overfitting to specific anatomical characteristics present in small clinical datasets. By encoding the governing physics into the loss function, PINN leverages self-supervision from the measured sinogram, ensuring that the learned reconstruction remains physically consistent even in out-of-distribution scenarios.

This approach is particularly advantageous for emerging imaging technologies like qRAI, where ground truth data from patients is either unavailable or impractical to obtain. Unlike purely data-driven networks (e.g., U-Net), PINN’s physics-constrained architecture enables robust performance in real-world conditions with sparse or noisy measurements. In our studies, PINN consistently outperformed both time-reversal and U-Net approaches across water tank, human phantom, and FLASH electron experiments—accurately recovering dose shape and magnitude, even with limited experimental training data.

The digital twin model plays a pivotal role in enabling quantitative reconstruction. Traditional RAI forward models often ignore real-world system complexities, leading to discrepancies between simulations and measurements. Our digital twin framework overcomes this by incorporating measured beam profiles, transducer impulse responses, and medium-specific acoustic properties—tuned to each experimental setup. With the digital twin, simulation data can now closely replicate experimental data, enabling networks trained on simulation data to be directly applicable to experimental setups. Although calibration requires upfront effort, once constructed, the digital twin remains stable and reusable across experiments, forming a foundation for reliable training data generation.

In our human torso phantom experiments, we demonstrated that PINN, trained from simulation and water tank data, can be directly applied to realistic anatomical geometries without retraining. This highlights its potential for clinical deployment, where acquiring diverse patient data for model training is infeasible. To address tissue heterogeneity and motion in future patient studies, we are developing a method to dynamically update the acoustic model using co-registered 3D ultrasound and planning CT. This will further improve robustness for *in vivo* applications during treatment^81^.

A key limitation remains the low signal-to-noise ratio (SNR) of radiacoustic signals, particularly in proton therapy where multiple beam pulses must be accumulated to produce usable images. This requirement currently limits temporal resolution. While FLASH electrons generate stronger signals due to higher per-pulse doses, 10 pulses are still needed to achieve optimal reconstruction. Although we have developed deep-learning-based denoising methods in prior work, their purely data-driven nature requires clinical datasets that are not yet available. A future direction is to integrate physics-informed denoising into the PINN workflow to enable single pulse imaging.

Importantly, the principles behind PINN are generalizable to other imaging modalities governed by physical models, such as ultrasound tomography^82^, X-ray computed tomography^83^, photoacoustic imaging^84,85^, and magnetic resonance imaging^86^. By embedding modality-specific physics into the network, PINN can serve as a flexible and powerful reconstruction engine across diverse applications, especially in settings where conventional supervised learning falls short due to data limitations.

In conclusion, qRAI powered by the PINN framework offers a transformative solution for precision dosimetry. It achieves quantitative, real-time dose reconstruction with minimal data requirements, supports in vivo imaging without labeled ground truth, and is extensible to a wide range of radiation and imaging modalities. Our experimental results confirm its accuracy in both proton and FLASH electron therapy and its compatibility with realistic anatomical settings. Looking ahead, we aim to further enhance performance through improved SNR, volumetric ultrasound integration, and expanded datasets that capture patient heterogeneity. With these developments, we envision qRAI as a cornerstone technology for adaptive radiotherapy, enabling online dose verification and ultimately improving patient safety and outcomes.

## Methods

### 3D RAI system

We evaluated the performance of the physics-informed neural network (PINN) framework using a radiacoustic imaging (RAI) system (Supplementary Fig. 5), integrated with a clinical radiotherapy machine capable of delivering either proton beams or FLASH electron beams. Radiacoustic signals were detected by a 256-element matrix ultrasound array, amplified, and processed through a custom 256-channel data acquisition system. To ensure precise synchronization, a trigger signal was generated using a photodiode coupled with a scintillator. This setup eliminates the need for mechanical scanning and enables real-time 3D radiacoustic imaging during radiation delivery. With the proton beam operating at a repetition rate of 750 Hz, the system achieves imaging rates of up to 75 frames per second using 10-signal averaging.

### Physics Model

This section outlines the theoretical foundations of the radiacoustic physics model integrated into our PINN framework, covering both the forward model of radiacoustic wave generation and the inverse reconstruction of the initial pressure distribution.

#### Radiacoustic Wave Generation and Propagation:

In quantitative radiacoustic imaging (qRAI), radiation energy deposition leads to a rapid local temperature rise, which in turn induces thermoelastic expansion and generates acoustic waves. Under the assumptions of thermal confinement and negligible acoustic attenuation, the wave equation governing pressure wave propagation is given by^87^:

(1) $$\left(\nabla^{2}-\frac{1}{c^{2}}\frac{\partial^{2}}{\partial t^{2}}\right)p(\vec{r},t)=\frac{1}{c^{2}}p_{0}(\vec{r})\frac{\partial \delta (t)}{\partial t}$$

where $p(\vec{r},t)$ denotes the acoustic pressure at location $\vec{r}$ and time *t, c* is the speed of sound, $p_{0}(\vec{r})$ is the initial pressure distribution, and *δ*(*t*) is a temporal distribution of $p_{0}(\vec{r})$, which in RAI, denotes the temporal profile of radiation pulse

The initial pressure $p_{0}(\vec{r})$ is proportional to the deposited dose, $D(\vec{r})$ and can be modeled as:

(2) $$p_{0}(\vec{r})=\Gamma \eta_{th}D(\vec{r})\rho$$

where *Γ* is the Grüneisen parameter, *η_th_* is the fraction of absorbed dose converted to heat, and *ρ* is the density of the irradiated medium.

The analytical solution for the pressure at a detection point ${\vec{r}}^{\prime }$ and time *t* is ^44^:

(3) $$p\left({\vec{r}}^{\prime },t\right)=\frac{1}{4\pi c^{2}}\frac{\partial }{\partial t}\left(\frac{1}{t}\int_{S(\vec{r},t)}p_{0}(\vec{r})dS^{\prime }(t)\right)$$

where *S*′(*t*) denotes a spherical surface defined by $|\vec{r}-{\vec{r}}^{\prime }|=\mathrm{ct}$. In compact operator notation, this relationship is expressed as^88^:

(4) $$p\left({\vec{r}}^{\prime },t\right)=M\left(p_{0}(\vec{r})\right)$$

where *M* represents the physical operator encompassing the forward process of acoustic wave generation and propagation.

#### Initial Pressure Reconstruction via Time-Reversal:

To reconstruct the initial pressure distribution $p_{0}(\vec{r})$, we apply a time-reversal (TR) method^68^, which numerically back-propagates the recorded acoustic signals in time. This inverse solution leverages the time-symmetry of the acoustic wave equation, allowing accurate recovery of the original pressure distribution:

(5) $$p_{tr}(\vec{r},t)=p_{m}(\vec{r},2T-t),\;T\leq t\leq 2T$$

where $p_{m}(\vec{r},t)$ is the measured pressure signal and *T* is the acquisition time. The initial conditions for solving the wave equation during back-propagation are:

(6) $$p_{tr}(\vec{r},T)=p_{m}(\vec{r},T),\;\frac{\partial p_{tr}(\vec{r},T)}{\partial t}=-\frac{\partial p_{m}(\vec{r},T)}{\partial t}$$

Solving the time-reversed wave equation with these conditions yields the reconstructed pressure distribution:

(7) $$p_{rec}(\vec{r})=\mathrm{TR}\left(p_{m}(\vec{r},t)\right)$$

where *TR* denotes the time-reversal operator. Importantly, incorporating prior knowledge of the acoustic properties of the medium—such as speed of sound, density, and attenuation—into the TR process enhances quantitative accuracy compared to standard universal back-projection (UBP) methods.

### Digital Twin Modeling

To accurately simulate the system-specific signal formation process in quantitative radiacoustic imaging (qRAI), we developed a digital twin^74,75^ model that incorporates the temporal characteristics of the radiation beam, the spatial integration effects of the ultrasound transducer, and the transducer’s impulse response. This comprehensive forward model enables the generation of realistic training data and serves as the physics engine embedded in the PINN framework.

The digital twin framework consists of four key components:

1. **Radiation Beam Temporal Profile**: As the first component of the digital twin, we captured the temporal profile of the radiation beam delivered by the radiotherapy system. A photodiode–scintillator assembly was placed directly beneath the beam to record its intensity output as a function of time. This temporal profile, denoted as *δ*(*t*), modulates the pressure waveform generated by the radiation energy deposition. Accordingly, the time-resolved pressure signal $p_{t}(\vec{r},t)$ at a detection point ${\vec{r}}^{\prime }$ is given by the convolution of the pressure output from the physical model $M(p_{0}(\vec{r}))$ with the radiation pulse profile:
(8) $$p_{t}\left({\vec{r}}^{\prime },t\right)=M\left(p_{0}(\vec{r})\right)⊗\delta (t)$$
  The measured pulse shapes are shown in Supplementary Fig. 1.
2. **Finite-Element Transducer Model**: Next, we account for the spatial averaging effect of the finite-sized transducer elements. Each transducer in the planar array has a physical size of 3×3 mm. To match the resolution of our image reconstruction, each element is subdivided into nine 1×1 mm sub-elements. The resulting pressure signal $p_{f}({\vec{r}}^{\prime },t)$ is calculated as the sum of the contributions from all sub-elements:
(9) $$p_{f}\left({\vec{r}}^{\prime },t\right)=\sum_{i}p_{t}\left({\vec{r}}^{\prime }+{\vec{d}}_{i},t\right)$$
  where ${\vec{d}}_{i}$ is the displacement vector from the center of the transducer element to the *i*th sub-element. This procedure is illustrated in Supplementary Figure 2.
3. **Transducer Impulse Response:** The final component of the digital twin model incorporates the frequency response of the ultrasound transducer (shown in Supplementary Figure 3), which behaves as a damped harmonic oscillator. The measured pressure signal $p_{m}({\vec{r}}^{\prime },t)$ is modeled as the convolution of the finite-element signal $p_{f}({\vec{r}}^{\prime },t)$ with the transducer’s impulse response, *IR*.
(10) $$p_{m}\left({\vec{r}}^{\prime },t\right)=p_{f}\left({\vec{r}}^{\prime },t\right)⊗IR$$
4. **Unified Forward Operator**: By combining all three components—the radiation pulse profile, finite-element spatial averaging, and transducer impulse response—we define a unified forward operator *F*, which represents the complete signal acquisition process in our digital twin system. Thus, the measured pressure signal can be expressed as:
(11) $$p_{m}\left({\vec{r}}^{\prime },t\right)=F\left(p_{0}(\vec{r})\right)$$

This digital twin framework provides a high-fidelity, end-to-end simulation of the qRAI system and plays a central role in training and validating the PINN model.

### Physics-Informed Neural Network

The core of our PINN framework is a 3D U-Net^89^ that maps the time-reversal reconstructed pressure map $p_{\mathrm{rec}}(\vec{r})$ to the estimated initial pressure distribution $p_{0}(\vec{r})$. This mapping is represented by a nonlinear function ***N(⋅)***, and the traditional learning objective for such a model is to minimize the L2 norm between the predicted and true initial pressure maps:

(12) $$p_{pred}=\underset{N}{argmin}\left({\left\|N\left(p_{\mathrm{rec}}\right)-p_{0}\right\|}_{2}^{2}\right)$$

To enforce physical consistency, we extend this formulation by integrating the forward physics operator *F*, which maps predicted pressure distributions to simulated sinograms. This leads to a dual-loss objective:

(13) $$p_{pred}=\underset{N}{\mathrm{argmin}}\left\{\lambda_{1}{\left\|N\left(p_{rec}\right)-p_{0}{\|}_{2}^{2}+\lambda_{2}F\left(N\left(prec\right)\right)-S_{m}\right\|}_{2}^{2}\right\}$$

where *λ*_1_ and *λ*_2_ are weighting parameters that control the trade-off between data fidelity and physics consistency. The model is trained by minimizing a composite loss function consisting of two terms:

- Pressure loss (image domain):
(14) $${ℒ}_{p}=\frac{1}{n}\sum |N(p_{\mathrm{rec}})-p_{0}{|}^{2}$$
- Sinogram loss (measurement domain):
(15) $${ℒ}_{S}=\frac{1}{n}\sum {\left|F\left(N\left(p_{rec}\right)\right)-S_{m}\right|}^{2}$$

This physics-informed formulation provides two key benefits: (1) it enables the network to generalize unseen data by learning from both image-level supervision and sinogram-level self-consistency, and (2) it reduces the dependency on large amounts of labeled data, which are difficult to obtain for emerging imaging modalities like qRAI.

### Pressure-to-Dose Calibration

Once the PINN reconstructs a quantitative pressure map, the final step is to convert this acoustic pressure into radiation dose distribution. While Eq. (2) provides a theoretical link between dose and pressure, the reconstructed pressure *p*_*pred*_ is relative in practice. This is because the acquired signals are measured in millivolts rather than Pascals and are affected by unknown amplifier gains within the acquisition system. To establish a pressure-to-dose relationship, we performed a calibration using a water tank experiment. We selected the Bragg peak location at 7 cm from the transducer as the calibration point, where the proton machine delivers 1.72 cGy per pulse (8 pC). The delivered dose at this location, *D*_*c*_, was extracted from the machine log files, and the corresponding reconstructed pressure value, *p*_*c*_, was obtained from the qRAI reconstruction. We then define the calibration factor *K* as:

(16) $$K=\frac{D_{c}}{p_{c}}$$

This scalar factor enables conversion of relative pressure values into quantitative dose by applying *K·p*_*pred*_ across the reconstructed dose map in the water tank setup.

For the human torso phantom, a direct pressure-to-dose conversion is more challenging because the absolute Grüneisen parameter (*Γ*) is unknown. Therefore, we performed a separate calibration using the same strategy. We selected the Bragg peak location at 5.2 cm depth, where the proton machine delivers a known dose of 1.88 cGy per pulse (8 pC). This approach allows for a relative calibration within the phantom geometry, enabling approximate dose estimation even in the absence of a precisely known tissue response.

### Evaluation Metrics

To quantitatively evaluate the accuracy of the reconstructed dose distributions, we employed three widely used image quality and dosimetric metrics: gamma index (γ), structural similarity index (SSIM), and peak signal-to-noise ratio (PSNR).

#### Gamma Index (γ):^90^

The gamma index $\gamma (\vec{r})$ was selected as the primary evaluation metric, as it is a standard criterion in radiotherapy for assessing both dose differences and spatial agreement between the planned and delivered dose. It combines distance-to-agreement (DTA) and dose difference into a single scalar value and is defined as:

(17) $$\gamma (\vec{r})=\text{min}\left[\sqrt{{\left(\frac{{\left\|\vec{r}-{\vec{r}}^{\prime }\right\|}_{2}}{\text{Δ}d}\right)}^{2}+{\left(\frac{{\left\|D_{GT}(\vec{r})-D_{pred}\left({\vec{r}}^{\prime }\right)\right\|}_{2}}{\text{Δ}D}\right)}^{2}}\right]$$

where:

- $D_{\mathrm{GT}}(\vec{r})$ is the reference (ground-truth) dose, $D_{\mathrm{pred}}({\vec{r}}^{\prime })$ is the predicted dose; $\vec{r}$ is the coordinate in $D_{\mathrm{GT}}(\vec{r})$ and ${\vec{r}}^{\prime }$ is the coordinated in $D_{\mathrm{pred}}({\vec{r}}^{\prime })$;
- Δ*d* is the distance-to-agreement (DTA) criterion (in mm) and Δ*D* is dose difference criterion (as a percentage of the reference dose).

A gamma value $\gamma (\vec{r})$ indicates that the reconstructed dose passes the specified acceptance criteria at location $\left(\vec{r}\right)$.

#### Structural Similarity Index (SSIM):

To assess structural fidelity, we used the structural similarity index (SSIM), which measures perceptual similarity between the reconstructed dose distribution and the reference dose. SSIM is defined as^91^:

(18) $$SSIM=\frac{\left(2\mu_{R}\mu_{I}+c_{1}\right)\left(2\sigma_{IR}+c_{2}\right)}{\left(\mu_{R}^{2}+\mu_{I}^{2}+c_{1}\right)\left(\sigma_{R}^{2}+\sigma_{I}^{2}+c_{2}\right)}$$

where:

- *I* and *R* are the reconstructed and reference dose images, respectively,
- *μ_R_* is an average of *R*, $\sigma_{I}^{2}$ is a variance of *I* and *τ_IR_* is a covariance of *I* and *R*.

There are two variables to stabilize the division such as *c*_1_ = (*k*_1_*L*)^2^ and *c*_2_ = (*k*_2_*L*)^2^. L is a dynamic range of the pixel intensities. *k*_1_ and *k*_2_ are constants by default *k*_1_ = 0.01 and *k*_2_ = 0.03.

#### Peak Signal-to-Noise Ratio (PSNR):

Lastly, PSNR was used to quantify the dose reconstruction fidelity. It is defined as^92^:

(19) $$PSNR=20\;{\text{log}}_{10}\left(\frac{MN\|G{\|}_{\infty }}{\|T-G{\|}_{2}}\right)$$

Where:

- *T* is the reconstructed dose image,
- *G* is the ground-truth dose image,
- *M* and *N* are the image dimensions (rows and columns),
- ∥*G*∥_∞_ is the maximum pixel valuein *G*.

Together, these metrics provide a comprehensive evaluation of both the spatial and dosimetric accuracy of our reconstructed dose maps.

## Supplementary Material

This is a list of supplementary files associated with this preprint. Click to download.

• Supplementary.pdf

## Figures and Tables

**Table 1.** Quantitative analysis of water tank data.

	TR	Unet	PINN
SSIM	0.508±0.111	0.823 ±0.035	0.947±0.019
PSNR	15.35±0.63	24.94±31.66	32.51±2.23
GI: 3mm/3%	0.090±0.077	0.562±0.136	0.880±0.089

SSIM: structural similarity index; PSNR: peak signal-to-noise ratio; GI: Gamma Index.

## Data Availability

The code supporting this study will be available via GitHub at https://github.com/SilverSunlyg/Quantitative-Dosimetry

## Data availability

The data supporting the findings of this study are provided within the Article and its Supplementary Information.
